# Somali, Latino, and Hmong Youth Perceptions of School Connectedness

**DOI:** 10.1089/heq.2021.0095

**Published:** 2022-07-04

**Authors:** April K. Wilhelm, Martha Bigelow, Mikow Hang, Luis E. Ortega, Shannon Pergament, Michele L. Allen

**Affiliations:** ^1^ Department of Pediatrics, Division of General Pediatrics and Adolescent Health, University of Minnesota, Minneapolis, Minnesota, USA.; ^2^ Program in Health Disparities Research, Department of Family Medicine and Community Health, University of Minnesota, Minneapolis, Minnesota, USA.; ^3^ Department of Curriculum and Instruction, College of Education and Human Development, University of Minnesota, Minneapolis, Minnesota, USA.; ^4^ SoLaHmo Partnership for Health and Wellness, Community University Health Care Center, Office of Clinical Academic Affairs, University of Minnesota, Minneapolis, Minnesota, USA.

**Keywords:** immigrant, adolescent, school environment, school connectedness, community-based participatory research

## Abstract

**Purpose:**

School connectedness positively influences adolescent health outcomes and is a key social determinant of health, yet, contributors to school connectedness for youth from immigrant communities remain poorly defined.

**Methods:**

This community-based participatory research study uses thematic analysis to identify contributors to Somali, Latino, and Hmong (SLH) adolescents' school connectedness. We conducted nine focus groups with 71 SLH male and female adolescents, the majority aged 13–18 years, in a United States Midwestern metropolitan area.

**Results:**

SLH students described contributors to their school connectedness that fit within three broad themes: (1) SLH students desire to be known and supported by their teachers as individuals, (2) specific teacher instructional approaches reinforce or undermine SLH student–school connections, and (3) transparency and fairness in school disciplinary practices are especially important for promoting Latino student–school connectedness.

**Conclusion:**

SLH youth perspectives offer ways for educators to foster increased school connectedness to improve academic and health outcomes among increasingly diverse student populations.

## Introduction

School connectedness, the process by which students build connections within school,^1^ strongly influences adolescent health.^2^ Higher levels of perceived school connectedness contribute to increased levels of academic success,^3^ reduced health risk behaviors,^4^ and an improved sense of mental wellbeing.^5,6^ Enhancing connectedness within diverse school systems requires a clear conceptualization of the contributors to school connectedness for all youth, yet, we know less about the salient factors for promoting school connectedness among youth from U.S. immigrant communities.^7–9^ Adolescents growing up within immigrant communities face increased risk of isolation and discrimination that can influence their identity development and health risk behaviors.^10^ Schools play important roles in creating welcoming school environments and reducing classroom bias for youth from immigrant backgrounds.^11^

This study uses a community-based participatory research (CBPR) approach that engages community members as equal partners in research^9,12^ to highlight Somali, Latino, and Hmong (SLH) student experiences within urban schools in one Midwestern city. We seek to elucidate ecological contributors to SLH student school connectedness to inform institutional reforms aimed at building connectedness among diverse populations of immigrant youth.

### Contributors to student school connectedness

Waters, Cross, and Runions describe one model of ecological influences on school connectedness, in which interpersonal (i.e., student–teacher relationships) and functional contributors (i.e., student-centered teaching practices and student perceptions of fair disciplinary systems) represent tangible targets for change.^13^ Higher levels of student–teacher relationship quality are associated with lower levels of risk behavior engagement,^14,15^ bullying,^16^ and depression symptoms,^14^ and higher levels of academic engagement and achievement.^3,17^ Functional contributors to school connectedness correlate with an increased sense of “being known” in school,^8^ higher reports of school connectedness,^18^ and fewer negative internalizing and externalizing behaviors.^19^

Strong school connections are associated with higher levels of academic achievement for students of color,^20^ yet, they are much less likely than white youth to rate their schools as safe^21^ or to report favorable relationships with their teachers^21^ or caring and equitable school relationships^22^—all potential disruptors to school connectedness. Furthermore, experiences of personal and family stressors, including exposure to violence and related psychological problem behaviors,^23^ can negatively influence students of color's level of school connection.^24^ Adolescents from U.S. immigrant communities, many who identify as students of color, face similar challenges^25^ that are compounded by limited school resources to support immigrant youth needs, such as native language and higher education preparation programs.^26,27^ Foreign-born youth drop out of high school at higher rates than their native-born peers, a disparity most pronounced among Latino youth.^28^

Studies of youth from immigrant communities support a role for positive student–teacher relationships in increasing academic engagement and performance.^29^ School connectedness may buffer Latino youth experiencing discrimination from dropping out of school^30^ and is associated with higher levels of academic engagement^25^ and lower levels of stress and despair^31^ among Asian immigrant students. School connectedness and climate are also strong predictors of adolescent substance use within Latino and Asian immigrant communities,^32–34^ indicating that initiatives to boost school connectedness may strengthen immigrant adolescent educational and health outcomes.

### Minnesota's diverse immigrant communities

Minnesota is currently home to the largest U.S. Somali diaspora, the second largest Hmong population, and a rapidly growing number of Latinos.^35^ Somali and Hmong youth from these Minnesotan communities are largely first and second generation immigrants, while Latino youth are increasingly second and third generation.^35^ Although these groups face common challenges with discrimination, poverty, and trauma,^36–38^ they also share assets, including strong intergenerational family support systems^39^ and a high value placed on educational achievement.^29^ Yet, persistent disparities in academic outcomes between white students and students from Minnesota immigrant communities^40^ underscore the need for a tailored educational approach.

This study focuses on the three largest ethnic immigrant groups in Minnesota—Somali, Hmong, and Latino—in which academic disparities remain unacceptably high. We examine SLH's school experiences to describe the school environmental characteristics that influence their school connectedness and to explore commonalities and differences in school experiences across groups.

## Methods

A CBPR team composed of three academic researchers and three community researchers from a CBPR research program of a large Midwestern federally qualified health center collaborated on this project. The partnership grew out of a shared vision among academic and community team members to use asset-based approaches to enhance the wellbeing of marginalized communities. Community researchers received formal CBPR and qualitative methodologic training and were involved in all stages of this research. A collaborative board, including parents and youth, teachers, and leaders from local community and educational organizations, provided further guidance.

### Participants

A total of 71 youth, including 20 Somali, 25 Hmong, and 26 Latino youth, participated in nine focus groups between September 2010 and August 2012 (Table 1). The mean age of participants was 15.7 years (with the majority between 13 and 18 years) and 49% were female. Participants included current students of a public middle or high school (grades 8–12) in the metropolitan area or former students at one of these schools during the prior academic year. Research team members collaborated with community agencies and schools to recruit SLH 8th through 12th graders through outreach to schools and after-school programs using word of mouth and flyers.

**Table 1.** Characteristics of Student Focus Group Participants

	Youth (*n*=71), %
Race/ethnicity	
African/African American/Somali	28
Asian American/Hmong	35
Hispanic/Latino	37
Gender	
Male	46
Female	49
Unknown	3
Age (years)	
13–15	42
16–18	55
Over 18	1
Grade	
8	11
9–10	46
11–12	34
Graduated within 6 months	4
Unknown	4

### Data collection

Trained bilingual research team members, all from SLH communities, conducted nine semistructured youth focus groups in English among ethnically similar mixed sex and age youth. Facilitators administered a demographic survey and then posed open-ended questions that fell within three broad domains: sources of youth resiliency; how teachers help students feel connected in schools; and how students would like teachers to better support them academically. Focus groups included six to nine participants, lasted an average of 69 min, and were audio recorded. Researchers obtained parental consent and youth assent for each participant. All participants received a $25 gift card. The researchers' Institutional Review Board approved the study protocol.

### Data analysis

Bilingual researchers transcribed the focus groups verbatim using NVivo 10 to capture codes and facilitate data management.^41^ The CBPR team analyzed the focus group transcripts using a version of the content-driven immersion-crystallization thematic analysis method adapted for participatory data analysis.^9,42^ The team's analytical approach allowed researchers to immerse themselves in the data, explore themes, and share cultural and experiential knowledge to incorporate multiple perspectives.^43^ Small groups composed of a community researcher who identified with one of the ethnic communities and an academic or community researcher not from one of these communities read and discussed transcripts to reach agreement on initial codes.

The entire team reviewed the codes before collaborating to create the final codebook and applying the coding scheme to the transcripts. The team collectively reviewed the coded transcripts, clarified coding discrepancies and discussed higher-order interpretation and organization of themes. Finally, a subset of team members drafted analytic summaries and reviewed them with the larger team to achieve consensus and to ensure trustworthiness of the analysis.

## Results

SLH youth's descriptions of how school environments influenced their school connectedness fit within three themes that describe the impact of teacher acknowledgment of and support for students' unique identities, teacher instructional approaches, and transparent school disciplinary practices. We examine these themes below and explore where youth across ethnicities agreed or differed in expressing how their school experiences related to connectedness.

### SLH students' desire to be known and supported as individuals

SLH students gravitated toward teachers who they felt attempted to get to know them as individuals with unique backgrounds and interests rather than as token members of their ethnic groups. Although most students acknowledged the challenges of bridging cultural and generational divides, they expressed a desire for teachers to engage in the relationship-building process and recognized these relationships as important to their school connectedness. Somali and Hmong students emphasized that even small gestures, such as asking about a student's interests outside the classroom (as opposed to specific knowledge of students' backgrounds), affirmed students' identities and facilitated positive student–teacher connections. Students in all groups described how teachers' personal stories of growth helped to forge student–teacher connections by enabling student understanding of teachers' vulnerabilities and resilience, thereby helping to boost students' self-confidence and future orientation.

Personal stories also demonstrated how teachers' lived experiences overlapped with their own, increasing teacher relatability:

This Puerto Rican principal…was just like, ‘you're Puerto Rican, I'm Puerto Rican. It's not easy for us out there and I understand sometimes you get mad and sometimes it's hard for you and it's different […] I'm going to help you out…But you got to settle down. You got to make sure you keep your cool […]’ And I was just like, dang. Like, that's respect. (L2002 Ref. 2)

All SLH students voiced challenges in connecting with teachers who they felt negatively stereotyped them, although their experiences with teacher biases differed. Somali youth often emphasized anti-Muslim or anti-immigrant biases. One Somali student (S3003 Ref. 1) recalled variable levels of teacher support of their outward expressions of faith: “On Fridays I wear a *qamis* (male Islamic tunic)…Some students call it ‘you're wearing a dress today. Why?’ Some teachers, they just laugh and sit there, and some teachers say, ‘it's a religious thing; it's not a dress.’” In contrast, Hmong and Latino youth predominantly described ethnic favoritism in class, ranging from perceptions of teacher preferences for white students to more explicit negative stereotyping. For example, Latino youth emphasized apparent ethnic bias in their descriptions of feeling labeled as “gang members [and] illegal immigrants” and described how teachers' personal prejudices even emerged during instructional activities:

He started talking about the Arizona law about deporting people […] He said if he was to go over there, nothing bad would happen to him and he's white, so obviously they wouldn't ask him for his papers or anything. He said, ‘There's no reason for people to get mad. If you're here illegally, you're committing a crime.’ So, at that point, I kind of lost respect for him. (L3003 Ref. 3)

Despite their different experiences of bias, SLH student internalization of these biases related to their cultural, geopolitical, or religious identities imperiled their evolving social identities in school, contributed to student disengagement, and reduced their desire and ability to foster connections with teachers.

### One size does not fit all: different instructional approaches reinforce or undermine SLH student–school connectedness

SLH students differed across groups in their preferred instructional approaches to promote their connections to school. Several Hmong and Latino students described teachers' enthusiasm and humor as important in promoting the accessibility of the lessons, but most Hmong students focused on the role of high academic expectations in maintaining their engagement and personal accountability. As one Hmong student (H2002 Ref. 1) recalled. “He makes you work hard for your grades. […] this teacher really makes you want to be motivate[d to] just come to school and like even though you don't like school, you come to school to be in [his] classroom.”

Rather than high expectations, most Somali students and a subset of Latino students expressed that individualized attention from teachers helped to boost their self-confidence and fuel their belonging. A Latino youth (L2002 Ref. 3) shared, “they're like, ‘we'll make time for you. Even if we're having class, we'll spend 10 minutes for you while the kids are doing work.’…They made me feel good about myself.”

Conversely, students from all backgrounds interpreted teachers not prioritizing their needs and rushing through material as the consequence of apathy toward students, teaching, or both. These students discussed their impressions that certain teachers approached their work as “simply a job” that was “just for the money,” sentiments articulated here by one Somali student (S1001 Ref. 1):

I don't think they want to be a teacher. They just do it for the money and hurry up to get things done…Say there are two minutes before the bell rings to go home and you're doing paperwork with the teacher. You're halfway there and you want to finish it. They're like ‘Hurry, hurry. Go. Go. I don't want to be late for somewhere.’

Teachers who the students felt did not prioritize time to help them struck many students as caring less about them personally, which undermined student motivation to learn from these teachers.

Finally, all SLH students described how feeling labeled as low achieving weakened their school connectedness. As one Hmong student (H1001 Ref. 1) shared: “they're more like ‘you don't need to worry, you're going to fail.’” Messages from teachers conveying expectations of student failure contributed to student disengagement. One Latino youth (L2002 Ref. 1) details how negative expectations impacted their school performance: “(T)he thing is, I knew I had the potential to do it, I just never really put my potential on the table for my teachers to see. Because every time, I would be like, ‘oh, I can do it.’ My teachers would be like, ‘no, you can't.’” Internalization of these negative messages led to many students questioning whether their academic efforts would pay off, fueling their disillusionment with school.

### Transparency and fairness in school disciplinary practices promote Latino student sense of belonging

Latino students were the only ones who commented extensively on school disciplinary systems. Latino students generally valued the role of school disciplinary systems in maintaining order, such as by holding students accountable for school attendance. One Latino student (L100 Ref. 2) shared their respect for teachers with strict, but transparent, classroom policies: “They don't bitch about anything. They don't like, ‘oh, I'm going to write you up.’ … He just says, ‘This is how it's done. Don't complain.’”

Yet, Latino youth also perceived teachers and administrators reprimanding them unfairly through ethnic profiling or overly punitive school disciplinary systems and expressed a strong preference for a transparent disciplinary system with uniform enforcement. One Latino student (L2002 Ref. 4) articulated how this contributed to their disengagement: “Too many administrators dismiss you, like, right away for, it could be like the dumbest things. … And it's just like, ‘Wow. You know I got like five more classes to go and I can still do a lot in one day.’”

While this student's portrayal of their interactions with an administrator lacks important contextual information, the scenario reflects a breakdown in communication and understanding between administrators and students that contributed to students feeling misjudged, disempowered, and poorly connected to school.

## Discussion

SLH youth's descriptions of their school experiences support the centrality of the student–teacher relationship, student-centered instructional approaches, and disciplinary policy transparency and fairness in shaping students' school connectedness. These perspectives provide a multicultural context to the school ecology literature,^13,18,44^ allowing for a deeper understanding of how a student's background and experiences contribute to their development of school connectedness, a key social determinant of health. Our findings highlight many similarities and a few differences between SLH student experiences.

SLH students voiced the importance of positive student–teacher relationships, echoing previous work in school connectedness.^3,13,14,17^ Most students in this study articulated previously reported desires for their teachers to learn about their individual backgrounds and interests by moving beyond “just teaching”^8^ and stereotypical classifications.^45^ While SLH students differed in their experiences of anti-immigrant, ethnic, and religious biases as previously described,^22,27,36^ their desire for teachers to get to know them as individuals resonates with previously voiced student perspectives on how small teacher gestures can forge positive student–teacher connections.^7,45^ In addition, generational differences may account for some of our observed differences in experiences and internalization of discrimination.^46,47^

SLH students also uniformly described how two functional components of school ecology—instructional approaches and transparent disciplinary systems^13^—influenced their school connectedness. Although their preferred instructional approaches varied by ethnic group, SLH students generally agreed with previous findings that teachers who conveyed expectations of lower achievement fueled student disengagement.^8,48^ SLH students perceived messages of lower expectations in teacher actions, such as when teachers did not prioritize time for individual questions as previously noted,^7,45^ along with more overt communications of expectations for student failure. Latino students were the only group to comment extensively on the disciplinary system in this study. Perhaps in response to feeling unfairly targeted in school based on ethnic stereotypes,^27^ Latino youth calls for transparent disciplinary systems aligned with previous voices from other immigrant communities.^19,44^

### Implications for practice

This research highlighted strong relationships with teachers as a fundamental factor in experiences of school connectedness among a diverse group of adolescents representing three distinct immigrant communities. Although adolescents differed in ways that they experienced barriers to developing positive relationships with their teachers, such as in experiences of bias and frustrations with explicitly stated low expectations, they largely shared themes that highlighted the ways in which urban immigrant adolescents experience discrimination and immigration-related stressors.^36–38^ Experiences described here by SLH youth of being the target of othering and labeled as low-achieving by their teachers due to their backgrounds are similar to those of other ethnically diverse groups of urban youth.^7,8^

Given the negative consequences of these experiences, which disrupt students' school relationships, sense of connectedness, and motivation, it is critical that schools address shared student-identified barriers to positive student–teacher relationships. Professional development that increases teacher knowledge about student backgrounds^27^ and emphasizes diversity as a classroom asset^49^ offer two evidence-based strategies to further support teachers' ability to build trusted relationships with diverse student populations. Incorporating classroom activities rooted in relational practices would also provide concrete opportunities for teachers to make impressions rooted in individual student identities rather than cultural stereotypes.^8^

Furthermore, interventions to help educators identify and reduce implicit biases^50^ and to positively reframe perceptions of minority students^51^ may decrease differential instructional and disciplinary treatment of students^52^ to foster connectedness. Although we acknowledge the challenging realities that teachers face within today's educational landscape in adapting their instruction to individual student's development and preferences,^53^ our findings support the importance of tailored, growth-mindset-based instructional approaches for youth from immigrant backgrounds.^54^ At the organizational level, school policies that recognize cultural and religious practices by allowing prayer in school and the use of religious or cultural garments can promote a welcoming environment for Muslim youth in particular.^27^

The results of this study shaped a teacher professional development curriculum to address these gaps and to strengthen student–teacher relationships and school connectedness.^55^ We have since integrated this professional development curriculum as one component within a multicomponent pragmatic participatory intervention trial that aims to promote policy and programmatic changes in schools to strengthen school connectedness as a social determinant for students who identify as Black, Indigenous, and People of Color;^56,57^ final results are forthcoming.

### Limitations

This study describes the school experiences of several U.S. adolescent subpopulations and provides insightful perspectives on opportunities to strengthen their school connectedness. However, our results represent the views of one urban population of SLH students and may not extend to other immigrant groups or SLH youth outside of this context. Future studies examining school connectedness should include students who have dropped out of the education system to provide a more holistic picture.

## Conclusions

This study details SLH youth perspectives on contributors to their school connectedness. Our findings highlight that, although differences in communication and teaching style preferences exist, SLH youth descriptions of how they develop school connections are quite similar. Youth emphasized the importance of strong, respectful student–teacher relationships and highlighted the functional facets of school ecology that promote their connectedness, such as being held to high expectations and a fair and transparent disciplinary system. The most striking message was the students' collective desire for an inclusive classroom, in which their unique identities are honored without being used as a meter of their ability or potential. Group differences in how students build trusting relationships and prefer to engage in the classroom offer insights for institutional reforms to foster school connectedness as a means of improving academic and health outcomes among youth from immigrant communities.

## Abbreviations Used

CBPR: community-based participatory research
HHS: U.S. Department of Health and Human Services
HRSA: Health Resources and Services Administration
SLH: Somali, Latino, and Hmong
